# Text Mining and Hub Gene Network Analysis of Endometriosis

**DOI:** 10.1155/2021/5517145

**Published:** 2021-12-07

**Authors:** Yinuo Wang, Songbiao Zhu, Chengcheng Liu, Haiteng Deng, Zhenyu Zhang

**Affiliations:** ^1^Department of Obstetrics and Gynecology, Beijing Chao-Yang Hospital Affiliated to Capital Medical University, Beijing 100020, China; ^2^Beijing United Family Hospital, Beijing 100015, China; ^3^MOE Key Laboratory of Bioinformatics, Center for Synthetic and Systematic Biology, School of Life Sciences, Tsinghua University, Beijing 100084, China; ^4^MOE Key Laboratory of Bioinformatics, Bioinformatics Division and Center for Synthetic and Systems Biology, BNRist, Department of Basic Medical Sciences, School of Medicine, Tsinghua University, Beijing, China

## Abstract

This study is aimed at systematically characterizing the endometriosis-associated genes based on text mining and at annotating the functions, pathways, and networks of endometriosis-associated hub genes. We extracted endometriosis-associated abstracts published between 1970 and 2020 from the PubMed database. A neural-named entity recognition and multitype normalization tool for biomedical text mining was used to recognize and normalize the genes and proteins embedded in the abstracts. Gene Ontology and Kyoto Encyclopedia of Genes and Genomes pathway enrichment analyses were conducted to annotate the functions and pathways of recognized genes. Protein-protein interaction analysis was conducted on the genes significantly cooccurring with endometriosis to identify the endometriosis-associated hub genes. A total of 433 genes were recognized as endometriosis-associated genes (*P* < 0.05), and 154 pathways were significantly enriched (*P* < 0.05). A network of endometriosis-associated genes with 278 gene nodes and 987 interaction links was established. The 15 proteins that interacted with 20 or more other proteins were identified as the hub proteins of the endometriosis-associated protein network. This study provides novel insights into the hub genes that play key roles in the development of endometriosis and have implications for developing targeted interventions for endometriosis.

## 1. Introduction

Endometriosis, defined as the appearance of endometrium-like tissue outside the uterus, is a chronic inflammatory disease with a high incidence and serious impact on the reproductive health of women [1, 2]. It is estimated that 10–15% of women of childbearing age suffer from endometriosis [3]. Extensive studies have been carried out to identify the genetic, epigenetic, and environmental factors involved in the development of endometriosis [4, 5]; however, the molecular mechanisms underlying the disease development remain elusive.

The number of biomedical articles published over recent decades continues to exponentially increase, as does the number of biomedical findings embedded in the text of published articles. With such explosive growth, it is very challenging to keep up with the latest research findings even in a specific research field. By applying algorithms and statistical methods, researchers can transfer the burden of dealing with massive information overload to computers, so that researchers can obtain the needed information from massive literature more effectively, which is the goal of text mining. To summarize and extract the most relevant information from massive publications and generate connections among different studies, text mining will introduce the recent progress and prospects for researchers who are interested in endometriosis and assist them to establish new hypotheses. The key step in text mining is biomedical-named entity recognition (BNER), which can be based on a dictionary or machine learning approach. The machine learning approach, which takes advantage of the underlying dictionary resource, is being widely used [6]. After the named entity recognition, text mining needs to normalize the recognized entities because a gene can be mentioned in different ways. Based on higher precision and recall rate, we used the neural BNER and multitype normalization tool (BERN) [7, 8] to systematically analyze the endometriosis-related articles.

Here, we systematically retrieved the endometriosis-associated abstracts from the PubMed database and mined the genes associated with endometriosis by using the BERN tool. Functional enrichment analysis provided a global view of the gene-hub regulation network associated with endometriosis.

## 2. Materials and Methods

Using the advanced search builder provided by PubMed, we searched for articles containing “endometriosis” within the title or abstract. We retrieved the PubMed Unique Identifier (PMID) for the searched articles published between January 1, 1970, and January 1, 2020, from the PubMed database using PubMed's Application Programming Interface (API) (https://www.ncbi.nlm.nih.gov/books/NBK25499/). Genes and proteins embedded in the abstracts of the articles were recognized using the BERN tool. A single gene can appear differently in the literature; hence, the recognized genes or proteins were normalized to the official gene symbol according to the National Center for Biotechnology Information's database for gene-specific information (Entrez gene database) [9].

The frequency was counted for each gene, and their probabilities of being cocited with endometriosis at a higher frequency were calculated. A false discovery rate- (FDR-) adjusted binomial test was used to determine the significance of each gene being cocited with endometriosis. Genes with *P* < 0.05 were considered significantly associated with endometriosis. Kyoto Encyclopedia of Genes and Genomes (KEGG) pathway enrichment analysis and Gene Ontology (GO) analysis, including molecular function (MF), biological process (BP), and cellular compartment (CC), were performed using the R package clusterProfiler [10]. NetworkAnalyst was used for the uterus tissue-specific protein-protein interaction network for endometriosis-associated genes [11]. The tissue-specific protein-protein interaction data were collected from the DifferentialNet (http://netbio.bgu.ac.il/diffnet/) database [12]. False discovery rate (FDR) < 0.05 was considered significant.

## 3. Results

A total of 21,633 PubMed Unique Identifiers (PMIDs) of articles were retrieved from PubMed. A total of 2259 genes were mentioned in the abstracts of endometriosis-related articles and were normalized with the Entrez gene database. A total of 433 genes were identified as endometriosis-associated genes with a *P* value < 0.05 calculated with binomial distribution. *GNRH1*, *PGR*, *TNF*, *CYP19A1*, and *IL6* were mentioned with the highest frequencies in endometriosis-related articles. The 20 most significant endometriosis-associated genes are listed in Table 1.

**Figure 1:**
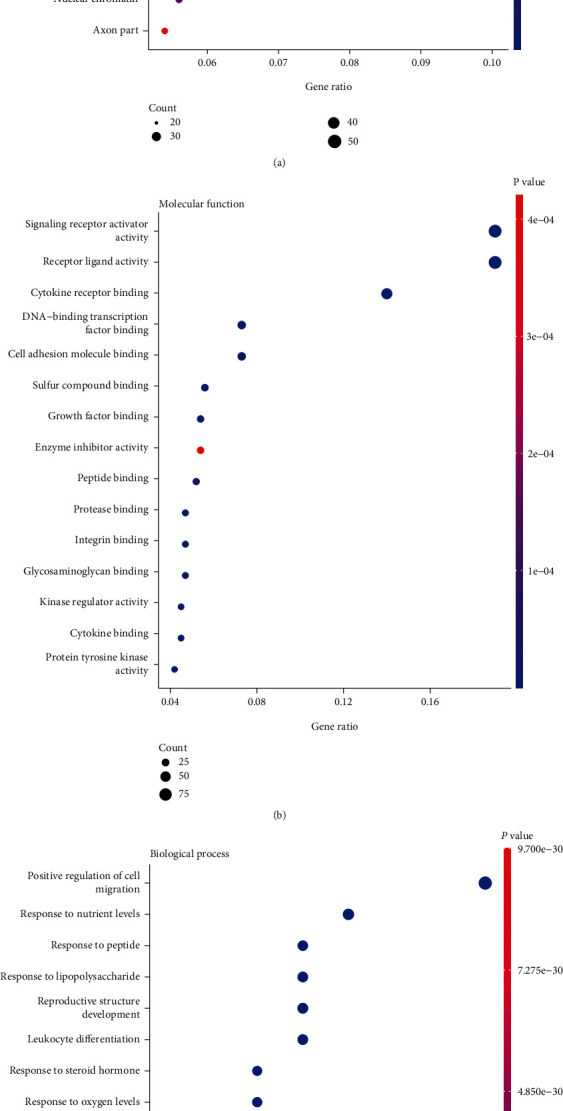
GO enrichment analysis of genes significantly cocited with endometriosis. The top 15 enriched terms of cellular compartment (a), molecular function (b), and biological process (c) are shown. The dot size represents the count of overlapped genes between the endometriosis-associated gene list and the total gene list of the given GO term. Gene ratio represented the count of overlapped genes for each GO item divided by the total number of genes in the endometriosis-associated gene list. The color scale represents the Bonferroni corrected *P* value. GO: Gene Ontology; CC: cellular components; MF: molecular function; BP: biological processes.

To understand the gene ontologies of the endometriosis-associated genes, GO enrichment analysis was performed. Most endometriosis-associated proteins are located in the intracellular as well as extracellular compartments. The intracellular compartment includes the cell membrane, cytoplasmic vesicle lumen, and nucleus, whereas the extracellular compartment includes the extracellular matrix and blood microparticles (Figure 1(a)). Most endometriosis-associated proteins perform receptor-ligand binding functions, including the binding between the receptors and cytokines, growth factors, steroids, and integrins. There are also proteins with functions downstream of receptor-ligand binding, including signal transducer activity, mitogen-activated protein kinase binding cascade, receptor tyrosine kinase activity, phosphatidylinositol-4,5-biphosphate 3-kinase activity, ubiquitin-like protein ligase binding activity, and transcription factor activity (Figure 1(b)). Consistent with the CC and MF analyses, the endometriosis-associated genes were mainly involved in biological processes, including cell migration regulation, reproductive structure development, leukocyte differentiation, and response to environmental factors such as lipopolysaccharides, peptides, oxygen, steroid hormones, and antibiotics (Figure 1(c)).

**Figure 2:**
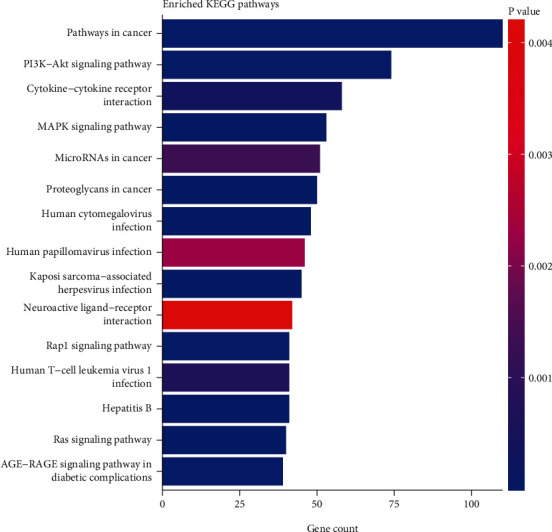
KEGG pathway enrichment analysis of genes which were significantly cocited with endometriosis. The top 15 significantly enriched KEGG pathways are presented. *X*-axis label represents pathway, and *Y*-axis label represents gene count. Gene count was the number of overlapped genes between the endometriosis-associated gene list and the total gene list of given KEGG pathway. The color of the bar represents the Bonferroni corrected *P* value. KEGG: Kyoto Encyclopedia of Genes and Genomes.

To clarify the pathways involved in the endometriosis-associated genes, KEGG pathway enrichment analysis was performed. Three pathway subclasses were enriched: signal transduction pathways, cancer-related pathways, and virus infection-related pathways. Signal transduction pathways included PI3K-Akt, AGE-RAGE, HIF1, and TNF signaling pathways. Cancer-related pathways included prostate cancer, colorectal cancer, pancreatic cancer, bladder cancer, and proteoglycans in cancer. Virus-infection-related pathways included hepatitis B virus and human cytomegalovirus infections (Figure 2).

**Figure 3:**
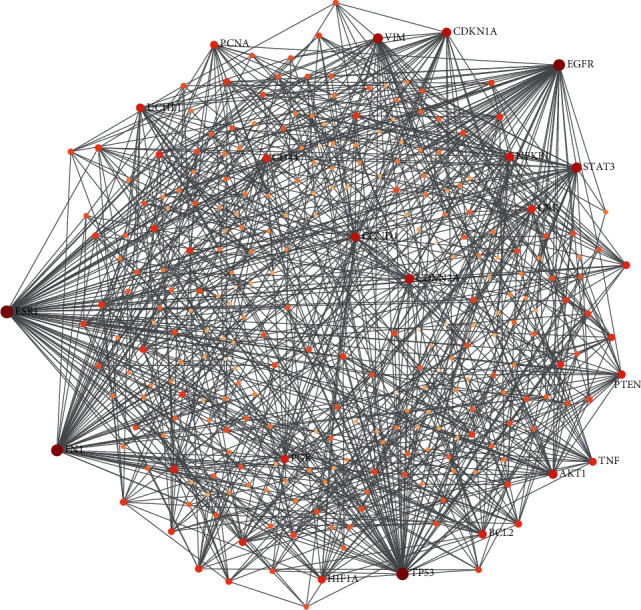
Uterus tissue-specific network analyses of endometriosis-associated genes by using NetworkAnalyst (http://www.networkanalyst.ca). A total of 100 genes that were mentioned more than 30 times were mapped to the uterus-specific molecular interaction database. The uterus-specific molecular interaction database was collected from the DifferentialNet database (http://netbio.bgu.ac.il/diffnet/). The mapping produced one big subnetwork with several smaller ones. Only subnetworks with at least 3 nodes are shown. The network was trimmed to minimum connected network by reducing less important nodes or edges to avoid too large and dense network. Each colored circle represents a protein interactor participating in the interaction network. The degree of a node is the number of connections it has to other nodes. The node size and color are proportional to the number of the degrees. Nodes with higher degree act as important “hubs” in the network.

To clarify the tissue-specific interaction relationship among the identified endometriosis-associated genes, the uterus tissue-specific protein-protein interaction network was established with the 100 genes that were mentioned with frequencies higher than 30. The network was trimmed to a minimum connected network, and 86 in 100 endometriosis-associated genes were seed proteins in the network. The network included 278 protein nodes and 987 protein-protein interaction links in total. There were 15 genes with the highest interaction links identified as the endometriosis-associated hub genes, including *ESR1*, *TP53*, *FN1*, *EGFR*, *STAT3*, *VIM*, *CDKN1A*, *CDKN2A*, *CCND1*, *NFKB1*, *AKT1*, *UCHL1*, *BCL2*, *PTEN*, and *PGR* (Figure 3).

**Figure 4:**
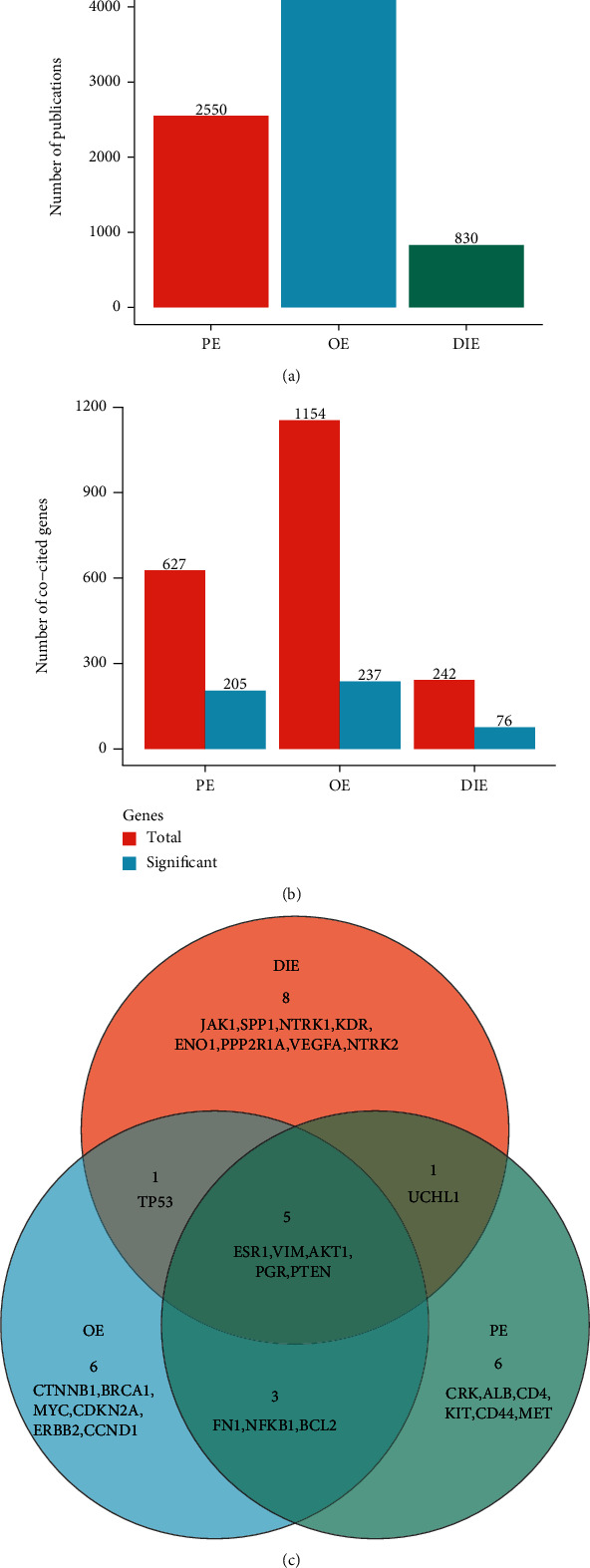
Text mining and hub gene analysis of three subtypes of endometriosis. (a) Bar plot of the number of the publications retrieved from PubMed by using “peritoneal endometriosis,” “ovarian endometriosis,” and “deep infiltrating endometriosis” as search terms. The publication date was set as from January 1, 1970, to January 1, 2020, in search input for PubMed. (b) Bar plot of the numbers of the genes appeared in the abstract of retrieved publications for each endometriosis subtype. The red bar represents the total genes that appeared in the endometriosis subtype-associated publications, while the blue bar represents the genes that were significantly cocited with the endometriosis subtype. False discovery adjusted binomial test was used to determine the significance of the gene being cocited with each subtype. (c) Venn diagram showing the common or endometriosis subtype-specific hub genes. Each circle represents one endometriosis subtype. The number in each area represents the number of hub genes which were shown in each intersect. PE: peritoneal endometriosis; OE: ovarian endometriosis; DIE: deep infiltrating endometriosis.

To investigate the specific hub genes for different subtypes of endometriosis including peritoneal endometriosis (PE), ovarian endometriosis (OE), and deep infiltrating endometriosis (DIE), the genes mentioned in the abstract were recognized and analyzed for each subtype separately [13]. “Peritoneal endometriosis” was used to retrieve 2550 articles from PubMed, while “ovarian endometriosis” or “deep infiltrating endometriosis” were used to retrieve 4538 and 830 articles, respectively (Figure 4(a)). The number of published articles associated with OE was highest, being 1.8-folds and 5.5-folds more than that associated with PE or DIE, respectively. Then, the BERN tool and Entrez gene database were used to recognize and normalize the genes embedded in the abstracts. A total of 627 genes were cocited with PE, while 1154 and 242 genes were cocited with OE or DIE, respectively (Figure 4(b)). The FDR-adjusted binominal test was used to filter the significant genes cocited with each subtype. A total of 205 genes were significantly associated with PE, while 237 and 76 genes were significantly associated with OE and DIE, respectively (Figure 4(b)). A uterus tissue-specific protein interaction network was conducted by NetworkAnalyst, and 15 hub genes were identified for each subtype (Figure 4(c)). There were 6 common hub genes among three subtypes, namely, *ESR1*, *VIM*, *AKT1*, *PGR*, and *PTEN*. PE subtype-specific hub genes included *CRK*, *ALB*, *CD4*, *KIT*, *CD44*, and *MET*. OE subtype-specific hub genes included *CTNNB1*, *BRCA1*, *MYC*, *CDKN2A*, *ERBB2*, and *CCND1*, while DIE subtype-specific hub genes included *JAK1*, *SPP1*, *NTRK1*, *KDR*, *ENO1*, *PPP2R1A*, *VEGFA*, and *NTRK2* (Figure 4(c)).

## 4. Discussion

Endometriosis has a serious impact on women's quality of life and imposes a substantial economic burden; however, there is poor understanding of the onset, diagnosis, and treatment of the disease. In this study, we established a dataset of endometriosis-associated genes by text mining of the published endometriosis-associated literature, annotated the functions and pathways, and identified 15 endometriosis-associated hub proteins. Some of the genes such as *PGR* and *ESR1* have been extensively investigated [14, 15], while the functions of a few genes have been less explored. Retrograde menstruation is a well-accepted hypothesis for endometriosis pathogenesis, in which viable endometrial fragments migrate and implant in other tissues of the pelvis [16, 17]. However, the detailed mechanisms of endometrial cells adhering to ovaries, their proliferation, acquiring blood supply, and resulting in endometriosis in some women remain elusive.

*STAT3, FN1, VIM, and EGFR*: these are migration factor coding genes, which provide insight into the regulatory roles of endometrial cells adhering to ovaries. STAT3 (signal transducer and activator of transcription 3) is a member of the STAT protein family, which are transcription factors expressed in response to cytokines and chemokines. Epithelial growth factor (EGF) can bind to the epithelial growth factor receptor (EGFR), which activates the receptor associated kinase, and STAT3 phosphorylation to form homo- or heterodimers and translocate into the nucleus. Activated STAT3 in the nucleus upregulates the migration related factors, including fibronectin 1 (encoded by *FN1*) and vimentin (encoded by *VIM*). Our results showed that epithelial-mesenchymal transition is a dominant process in endometriosis, consistent with previous reports [18, 19].

*NFKB1*: this gene encodes the NFKB protein, which is a transcription regulator activated by different stimuli such as cytokines and lipopolysaccharide [20]. Activated NFKB upregulates the expression of genes involved in the immune response, cell growth, and apoptosis [21, 22]. Since dysregulated activation of NFKB results in inflammatory diseases, our results indicate that inflammation plays a role in endometriosis.

*UCHL1*: the ubiquitin proteasome pathway (UPP) is essential for removing proteins within endometrial cells. The *UCHL1* gene encodes the ubiquitin C-terminal hydrolase L1, which is a thiol protease that hydrolyzes a peptide bond at the C-terminal glycine of ubiquitin. The *UCHL1* gene is highly expressed in the neurons and cells of the diffuse neuroendocrine system. It has been reported that *UCHL1* dysfunction is associated with many neurodegenerative diseases [23], while there are few reports on *UCH1* and endometriosis [24].

An excellent paper by Cui et al. integrated the existing profiling data of ectopic endometrium (EC), eutopic endometrium (EU), and normal endometrial (NE) tissues and identified the common and endometrial tissue-specific differentially expressed genes (DEGs) by using R package limma [25]. They identified 394 core differential genes (CDGs) and 5 hub genes from the protein-protein-interaction (PPI) network including *PIK3CA*, *CDK1*, *BIRC5*, *KIF2C*, and *ITGA1*. *ESR1* was one of the CDGs and interacted with the hub gene *PIK3CA*, which was also identified as the hub gene in our study, while other hub genes from our study were not identified as CDGs in Cui's paper. The other excellent paper by Jiang et al. integrated four publicly existing microarray datasets including 1 dataset for peritoneal endometriosis (PE), 2 datasets for ovarian endometriosis (OE), and 1 dataset for deep infiltrating endometriosis (DIE) and used the R package limma to identify common and endometriosis subtype-specific DEGs [26]. Jiang et al. found that the PI3K-Akt signaling pathway was significantly enriched for OE-specific DEGs. *AKT1* was one of common hub genes among 3 endometriosis subtypes in our study, indicating that the outcome of text mining was partially consistent with DEG analysis, but it also provided some unique findings.

The limitations of the present study were the following. Only 2259 genes mentioned in the abstract were analyzed. Text mining with the greater access to the full text of publications may allow us to gain more information. Text mining is an effective tool for generating new hypothesis. And future experimental validation is needed to confirm the relationship among the identified hub genes.

## 5. Conclusions

Our study outcomes provide a global view on the functions and pathways of the genes reported in endometriosis-associated literature. This systematic study provides novel insights into the pathogenesis and targeted therapies for endometriosis. However, since the results are based on the text mining of previously published literature, further experimental exploration and validation are required to better understand the functions of the hub genes in endometriosis.

## Figures and Tables

**Table 1:** The 20 most significant endometriosis-associated genes.

Gene ID†	Symbol	Description	Frequency‡	*P* value
2796	*GNRH1*	Gonadotropin releasing hormone 1	859	<0.001
5241	*PGR*	Progesterone receptor	522	<0.001
7124	*TNF*	Tumor necrosis factor	480	<0.001
1588	*CYP19A1*	Cytochrome P450 family 19 subfamily A member 1	446	<0.001
3569	*IL6*	Interleukin 6	444	<0.001
7422	*VEGFA*	Vascular endothelial growth factor A	357	<0.001
94025	*MUC16*	Mucin 16, cell surface associated	350	<0.001
3553	*IL1B*	Interleukin 1 beta	322	<0.001
2099	*ESR1*	Estrogen receptor 1	295	<0.001
6046	*BRD2*	Bromodomain containing 2	254	<0.001
5351	*PLOD1*	Procollagen-lysine,2-oxoglutarate 5-dioxygenase 1	231	<0.001
268	*AMH*	Anti-Mullerian hormone	224	<0.001
4318	*MMP9*	Matrix metallopeptidase 9	174	<0.001
2069	*EREG*	Epiregulin	169	<0.001
5743	*PTGS2*	Prostaglandin-endoperoxide synthase 2	156	<0.001
4313	*MMP2*	Matrix metallopeptidase 2	144	<0.001
7157	*TP53*	Tumor protein p53	131	<0.001
5617	*PRL*	Prolactin	131	<0.001
6347	*CCL2*	C-C motif chemokine ligand 2	127	<0.001
4790	*NFKB1*	Nuclear factor kappa B subunit 1	127	<0.001

^†^Gene ID: accession number from the ENTREZ gene database. ^‡^Frequency: the frequency of each gene being mentioned in endometriosis-associated publications.

## Data Availability

The endometriosis-associated abstract data used to support the findings of this study are available from the corresponding authors upon request.
